# Difference in the prevalence of hypertension and its risk factors depending on area-level deprivation in Japan

**DOI:** 10.1186/s13104-022-05931-6

**Published:** 2022-02-10

**Authors:** Tasuku Okui, Jinsang Park

**Affiliations:** 1 Medical Information Center, Kyushu University Hospital, Maidashi3-1-1 Higashi-ku, Fukuoka, Fukuoka 812-8582 Japan; 2 Department of Pharmaceutical Sciences, International University of Health and Welfare, Fukuoka, Japan

**Keywords:** Japan, Hypertension, Obesity, Smoking, Alcohol drinking

## Abstract

**Objectives:**

Area-level deprivation is an important factor related to mortality or health behaviors; however, a study investigating differences in hypertension prevalence depending on area-level deprivation has not been conducted in Japan. We investigated differences in the prevalence of hypertension and its risk factors, i.e. obesity, smoking, alcohol consumption, and heavy alcohol drinking depending on area-level deprivation using nationwide health checkups data in 2018.

**Results:**

Area-level deprivation was derived from census data. An analysis of the data by secondary medical areas revealed that the age-standardized proportions of individuals whose systolic blood pressure was ≥ 140 mmHg, those whose diastolic blood pressure was ≥ 90 mmHg, those whose body mass index was ≥ 25 or 30 kg/m^2^, smokers, and heavy alcohol drinkers showed an increasing trend with an increase in the deprivation level. The relative index of inequality, which can be interpreted as the ratio of the age-standardized proportion for the most deprived area compared with that for the least deprived area, was significantly greater than 1 for all proportions, except for the proportion of drinkers in women. Overall, there was a disparity in the prevalence of hypertension and its risk factors depending on area-level deprivation.

**Supplementary Information:**

The online version contains supplementary material available at 10.1186/s13104-022-05931-6.

## Introduction

Hypertension is a major risk factor for cardiovascular diseases and one of the leading causes with a significant effect on mortality in Japan [1]. Mean systolic and diastolic blood pressures are shown to have decreased over recent years in Japan [2]. However, the current prevalence of hypertension is approximately 30% and 20%, respectively, for men and women aged 20 or more years in Japan [2]. Preventing occurrence of hypertension is still an important public health concern in Japan.

Several studies have shown that there are regional differences in the prevalence of hypertension in Japan [3, 4]. One such study revealed that the number of steps taken by an individual was significantly associated with prevalence [3]. However, a study investigating regional differences in hypertension prevalence depending on area-level deprivation has not been conducted in Japan. In other countries, area-level deprivation or socioeconomic position is shown to be significantly associated with regional hypertension prevalence [5, 6]. In Japan, stroke incidence or all-cause mortality is shown to be affected by area-level deprivation [7, 8]. Some behaviors, such as unhealthy dietary habits, were also shown to be associated with area-level deprivation in epidemiological studies [9, 10]. Therefore, it is important to verify the disparity in hypertension prevalence. In addition, factors such as obesity, smoking, and alcohol drinking are known to be risk factors for hypertension in Japan [11–16], and these risk factors for hypertension might also vary depending on area-level deprivation. However, an association between these factors and area-level deprivation has not been investigated using nationwide government statistics in Japan. If an association between area-level deprivation and prevalence of hypertension and its risk factors exists, we might be able to conduct administrative preventive measures focusing on those areas.

Here, we investigated differences in proportions of hypertension and its risk factors depending on area-level deprivation using specific health checkup data in Japan.

## Main Text

### Methods

The Ministry of Health, Labor, and Welfare in Japan began performing specific health checkups and providing specific health guidance from 2008 [17]. Insured persons and their dependents aged 40–74 years are encouraged to receive a medical checkup concerning lifestyle-related diseases every year [17, 18]. If a person has a high possibility of developing a lifestyle-related disease, they receive specific health guidance. We used data on results of the checkups by five-year age groups, sex, and secondary medical areas in 2018 [19]. Specifically, we used data on systolic blood pressure (BP), diastolic BP, body mass index (BMI), smoking status, alcohol drinking status, and amount of alcohol drinking for the analysis. BP and BMI are measured in the checkups, while smoking and alcohol drinking status are self-reported by questionnaires. The secondary medical area is a unit of regions which is smaller than a prefecture, but is larger than a municipality [20]. In addition, map data of Japan were obtained from a government website [21]. We used the specific health checkup data conducted by the government, and sample size estimation was not conducted for this study.

BMI $\ge$ 25 kg/m^2^ was categorized as obesity, and systolic BP $\ge$ 140 and diastolic BP $\ge$ 90 were each categorized as hypertension [22]. According to the criteria proposed by the World Health Organization, BMI $\ge$ 30 kg/m^2^ was categorized as obesity [23], whereas BMI $\ge$ 25 kg/m^2^ has been proposed as the criterion for obesity in Asian and Japanese people [24, 25]. We also showed the results of BMI $\ge$ 30 kg/m^2^ in addition to BMI $\ge$ 25 kg/m^2^ for reference. Regarding alcohol drinking status, a participant is asked in a questionnaire the frequency of drinking alcoholic beverages. The choices are “every day,” “sometimes,” and “not drink.” We categorized “everyday” and “sometimes” as drinkers. In another questionnaire, a participant is asked about alcohol drinking amount per drinking day based on gou (unit of sake). Annotation of the questionnaire explains 1 gou (180 ml of sake) is approximately equivalent to: 500 ml of beer, 110 ml of syochu, 60 ml of whiskey, or 240 ml of wine. The answer choices are “Under 1 gou’’, “1–2 gou’’, “2–3 gou’’, and “3 gou or more’’. We defined 2 gou or more for men and 1 gou or more for women per drinking day as heavy drinkers because these alcohol consumptions are shown to be related to hypertension incidence in Japan [15].

Area-level deprivation was calculated based on a method proposed in a previous study [8], which has been used for several studies [7, 9]. The area-level deprivation was derived based on the following equation using characteristics available in the Census data, and a detailed explanation has been provided in a previous study [8].

$$ Area \, level \, deprivation \,= \,2.99 \times \, proportion \, of \, old \, couple \, households\, + \,7.57\, \times \, proportion \, of \, old \, single \, households \, + \,17.4 \, \times \, proportion \, of \, fatherless \, households \, +\, 2.22\, \times \, proportion \, of \, rent \, houses \,+ \,4.03 \,\times\, proportion \, of \, sales \, and \, service \, workers \,+ \,6.05\, \times \, proportion \, of \, agricultural \, workers \,+ \,5.38 \, \times \, proportion \, of \, blue{-}collar \, workers \, + \,18.3 \, \times \, unemployment \, rate $$

The Census data in 2015 was used in this study because the most recent data currently available was that of 2015 [26, 27].

We classified the secondary medical areas by area-level deprivation into 5 quintiles, and showed them in a map of Japan. In addition, we calculated age-standardized proportions of each of the outcomes by sex and the quintiles, by using age-group specific number of participants in all of Japan as a standard population. Moreover, we calculated slope index of inequality (SII) and relative index of inequality (RII) for age-standardized proportions of each of the outcomes. SII and RII are often used for quantifying degree of health disparities [28–30]. 95% confidence interval (CI) for RII was calculated based on bootstrap method. All statistical analyses were conducted using R3.6.3 (https://www.R-project.org/).

### Results

Additional file 1: Table S1 shows basic characteristics of variables used in this study for the 335 secondary medical areas.

**Fig. 1:**
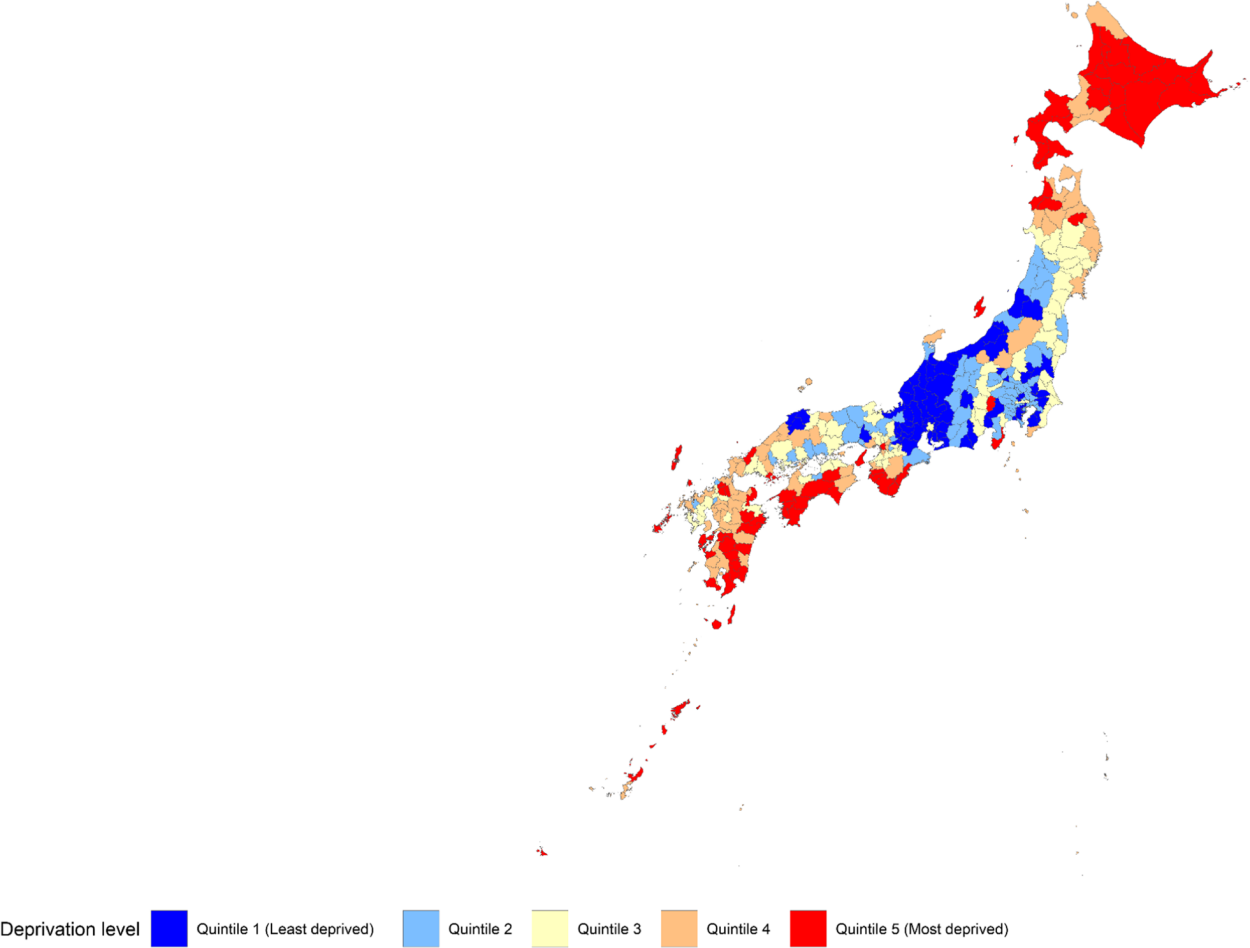
Geographic distribution of area-level deprivation in Japan. The figure was generated by the authors using open data from government websites, and is exempt from copyright issues

Figure 1 shows a map of area-level deprivation in Japan. The most deprived areas tended to be observed in Hokkaido and Okinawa; whereas, the central parts are less likely to be deprived.

Additional file 1: Table S2 shows the number of participants for each type of measurement by deprivation level. The number of participants tends to be smaller in more deprived areas because populations tend to be smaller in deprived areas.

Additional file 1: Table S3 shows sex and age-group specific proportion of each of the outcomes in all of Japan. The proportions largely varied depending on age groups, suggesting an analysis adjusting for age is important.

Table 1 shows age-standardized proportions of the outcomes by the area-level deprivation by sex. The proportion showed an increasing trend as the deprivation level increased in most of the outcomes.

**Table 1:** Age-standardized proportions of the outcomes by the area-level deprivation by sex

Variable	Area-level deprivation				
	Quintile 1 (Least deprived areas)	Quintile 2	Quintile 3	Quintile 4	Quintile 5 (Most deprived areas)
Men					
Proportion of persons whose systolic BP ≥ 140 (mmHg)	18.3	18.9	19.4	20.2	21.4
Proportion of persons whose diastolic BP ≥ 90 (mmHg)	15.9	16.7	16.7	16.9	17.4
Proportion of persons whose BMI ≥ 25 (kg/m^2^)	33.6	34.7	35.1	36.7	37.6
Proportion of persons whose BMI ≥ 30 (kg/m^2^)	5.6	5.9	5.9	6.4	6.7
Proportion of smokers	32.5	33.1	33.8	35.2	36.5
Proportion of drinkers	69.3	69.8	69.8	70.8	70.7
Proportion of heavy alcohol drinkers	23.6	24.4	24.3	24.4	26.2
Women					
Proportion of persons whose systolic BP ≥ 140 (mmHg)	14.1	14.4	14.6	14.8	15.9
Proportion of persons whose diastolic BP ≥ 90 (mmHg)	7.2	7.7	7.4	7.4	7.7
Proportion of persons whose BMI ≥ 25 (kg/m^2^)	19.2	20.2	20.5	21.7	23.1
Proportion of persons whose BMI ≥ 30 (kg/m^2^)	3.8	4.1	4.2	4.5	5.0
Proportion of smokers	8.7	9.9	9.7	10.4	13.0
Proportion of drinkers	40.9	42.7	41.6	42.0	41.8
Proportion of heavy alcohol drinker	24.3	24.7	25.2	25.9	27.8

*BMI* body mass index, *BP* blood pressure

Table 2 shows the SII and RII for the outcomes. A significant disparity dependent on the area-level deprivation was observed regardless of sex and the outcomes, except for the proportion of drinkers in women.

**Table 2:** Degree of the disparity in the outcomes depending on area-level deprivation

Variable	Men		Women	
	SII (95% CI)^a^	RII (95% CI)^b^	SII (95% CI)^a^	RII (95% CI)^b^
Proportion of persons whose systolic BP ≥ 140 (mmHg)	3.36 (2.46, 4.26)	1.18 (1.13, 1.23)	2.04 (1.29, 2.78)	1.14 (1.09, 1.20)
Proportion of persons whose diastolic BP ≥ 90 (mmHg)	1.66 (0.88, 2.44)	1.10 (1.05, 1.15)	0.45 (0.03, 0.87)	1.06 (1.00, 1.13)
Proportion of persons whose BMI ≥ 25 (kg/m^2^)	6.01 (4.95, 7.08)	1.19 (1.15, 1.22)	5.26 (4.22, 6.29)	1.28 (1.22, 1.34)
Proportion of persons whose BMI ≥ 30 (kg/m^2^)	1.52 (1.15, 1.90)	1.29 (1.21, 1.38)	1.37 (1.05, 1.70)	1.37 (1.28, 1.48)
Proportion of smokers	4.52 (3.45, 5.60)	1.14 (1.10, 1.17)	3.50 (2.60, 4.40)	1.44 (1.30, 1.58)
Proportion of drinkers	2.81 (1.59, 4.02)	1.04 (1.02, 1.06)	0.70 (-0.98, 2.38)	1.02 (0.97, 1.06)
Proportion of heavy alcohol drinkers	3.20 (1.74, 4.67)	1.15 (1.08, 1.23)	4.37 (2.50, 6.23)	1.20 (1.11, 1.30)

*CI* confidence interval, *SII* Slope index of inequality, *RII* Relative index of inequality, *BMI* body mass index, *BP* blood pressure

^a^SII can be interpreted as the difference of age-standardized proportion between the most deprived area and that for the least deprived area

^b^RII can be interpreted as the ratio of age-standardized proportion for the most deprived area compared with that for the least deprived area

### Discussion

We showed that prevalence of hypertension and its risk factors was different depending on area-level deprivation. Here we discuss possible reasons for the results and implications.

An association between regional deprivation level and obesity prevalence was shown for the first time in Japan; whereas, it was known that regional differences in average income level, educational level, or unemployment rate was related to obesity prevalence [31, 32]. In other developed countries, an association between area-level deprivation and obesity prevalence has been shown [33–35]. The lack of healthy food or the lack of recreational infrastructure has been pointed as possible causes [33, 36, 37]. An association between low socioeconomic status or area-level deprivation and unhealthy dietary habits are also shown in Japan [10, 38, 39]. Therefore, the unhealthy dietary habits are possible causes of the associations. In contrast, globally, obesity is not necessarily negatively associated with low socioeconomic status. In low- and middle-income countries, the prevalence of overweight or obesity tended to increase with wealth [40].

Smoking is another major risk factor for hypertension [11, 41] and a risk factor for cardiovascular diseases in Japan [42]. An association between smoking and low socioeconomic status has been shown in many studies [43, 44]. Possible reasons for an association between low socioeconomic status and smoking are that persons with low socioeconomic status are less frequently exposed to public health campaigns or that opportunities for smoking may still be greater in low socioeconomic status groups [45]. Additionally, it is considered that there are regional differences in policies for no smoking in restaurants or workplaces, and regional differences might also exist for a number of clinics conducting treatment for smoking cessation. Those factors may vary depending on area-level deprivation.

Proportion of heavy alcohol drinking was related to area-level deprivation both in men and women, whereas, prevalence of alcohol drinkers was not associated with it in women. Alcohol drinking is known to be positively related to high educational level; however, problematic or heavy alcohol drinking is related to lower educational level in Japan [46, 47]. Therefore, it is not surprising that proportion of alcohol drinkers did not differ depending on area-level deprivation. A possible reason for an association between heavy alcohol drinking and individual socioeconomic status in Japan can be due to education increasing understanding of the negative effects of heavy alcohol drinking [47]. Also, an association between heavy alcohol drinking and area-level deprivation was shown in studies in other countries [48, 49], and an increased alcohol outlet density in deprived areas was pointed out as a factor [48]. In contrast, according to a study carried out in the United States, people in the least deprived neighborhoods were most likely to be the heaviest alcohol drinkers [50], and a survey carried out in the U.K. revealed that people in the most deprived neighborhoods were less likely to report excess consumption in comparison with those in the least deprived ones [51]. Therefore, the relationship between alcohol drinking and neighborhood deprivation varies from one country to another.

Other factors, such as dietary habits and treatment rate are also considered to be related to the regional disparity in hypertension prevalence. Participation rate of health checkups is affected by income level in Japan [38]. Therefore, it is possible that detection of hypertension occurs late for persons with low socioeconomic status. In addition, low income level is also known to be associated with decreased access to outpatient care [52], and treatment status of hypertension might be also affected by socioeconomic status. Salt intake is another major factor related to the trend of hypertension prevalence in Japan [53], with salt intake also being affected by socioeconomic status [12, 54].

There are people who are not participating in the specific health checkups, such as non-insured persons, and detecting and treating hypertension patients, including the non-participants, early in the deprived areas is important. In addition, it is important to assess differences in environments affecting health behaviors depending on area-level deprivation, such as number of alcohol outlets, smoking areas, clinics conducting medical care for smoking cessation, and public infrastructure for physical activity. Moreover, there are some common fundamental causes for the disparity in those risk factors, such as low educational level and low income. It is considered that people in deprived areas are mutually affected by unfavorable health behaviors of people in those areas. Therefore, massive health education and health checkups targeting all of the citizens from childhood is considered to be particularly important in the deprived areas.

### Limitations

Firstly, participation in the specific health checkups is not mandatory, and participation rate is a little higher than 50% [17]. Therefore, differences in participation rates among regions might have affected the results of this study. Secondly, smoking and alcohol drinking status is based on self-reported questionnaires, which may have led to certain inaccurate responses. Thirdly, we could obtain data on average alcohol consumption per drinking day and frequency of alcohol drinking. However, data on alcohol consumption per day could not be obtained, but the amount of daily alcohol consumption is often an important factor for hypertension incidence [14]. Publication of data on combination of frequency of alcohol drinking and average alcohol consumption per day is warranted for the specific health checkups data. Lastly, there are multiple types of methods for deriving area-level deprivation level, and we used one of those methods. Confirming disparity using multiple kinds of area-level deprivation levels might be needed for verifying the robustness of these results.

## Supplementary Information

**Additional file 1: Table S1**. Basic characteristic of variables used in this study for the 335 secondary medical areas. **Table S2**. Number of participants for each type of measurement by deprivation level. **Table S3**. Sex and age group–specific proportion of each of the outcomes in all of Japan.

## Data Availability

The specific health checkups data were available from: https://www.mhlw.go.jp/stf/seisakunitsuite/bunya/0000177221_00010.html. The Census data other than data on the proportion of households living in rental housing are available from: https://www.e-stat.go.jp/regional-statistics/ssdsview. Data on the proportion of households living in rental housing were obtained from: https://www.e-stat.go.jp/stat-search/files?page=1&toukei=00200521&result_page=1. Map data by the secondary medical areas in Japan were obtained from the digital national land information of the Ministry of Land, Infrastructure, Transport and Tourism, and were available from: https://nlftp.mlit.go.jp/ksj/gml/datalist/KsjTmplt-A38-v2_0.html. We generated Fig. 1 by processing the map data, and the following R packages were used: sf (http://cran.nexr.com/web/packages/sf/sf.pdf), ggplot2 (https://cran.r-project.org/web/packages/ggplot2/ggplot2.pdf), RColorBrewer (https://cran.r-project.org/web/packages/RColorBrewer/RColorBrewer.pdf), and ggthemes (https://cran.r-project.org/web/packages/ggthemes/ggthemes.pdf).

## Abbreviations

CI: Confidence interval
SII: Slope index of inequality
RII: Relative index of inequality
BMI: Body mass index
BP: Blood pressure
