# Supplementary material for: Difference in the prevalence of hypertension and its risk factors depending on area-level deprivation in Japan
Source: BMC Res Notes. 2022 Feb 10;15:37. doi: 10.1186/s13104-022-05931-6 (PMC8832789; doi:10.1186/s13104-022-05931-6)
Supplement: Supplementary file 1 — Additional file 1: Table S1. Basic characteristic of variables used in this study for the 335 secondary medical areas. Table S2. Number of participants for each type of measurement by deprivation level. Table S3. Sex and age group–specific proportion of each of the outcomes in all of Japan. [file 13104_2022_5931_MOESM1_ESM.docx]

Supplementary table 1. Basic characteristic of variables used in this study for the 335 secondary medical areas.

| Characteristics | Median (Interquartile range) |
| --- | --- |
| Regional characteristics |  |
| Proportion of older couple households | 12.8 (11.3 - 14.7) |
| Proportion of older single households | 11.5 (9.8 - 14.3) |
| Proportion of fatherless households | 1.5 (1.3 - 1.7) |
| Proportion of households with rental housing | 24.8 (19.6 - 31.1) |
| Proportion of sales and service workers | 10.4 (9.3 - 12.1) |
| Proportion of agriculture, forestry, and fisheries workers | 5.4 (2.4 - 10.5) |
| Proportion of blue–collar workers | 7.1 (6.5 - 7.8) |
| Proportion of unemployed persons | 4.1 (3.5 - 4.6) |
| Number of male participants or respondents |  |
| BMI measurement | 25975 (11344 - 62019) |
| Systolic BP measurement | 25990 (11344 - 61986) |
| Diastolic BP measurement | 25988 (11338 - 61984) |
| Smoking status | 26002 (11364 - 61997) |
| Alcohol drinking status | 23934 (10588 - 58170) |
| Amount of alcohol consumption per drinking day | 19429 (8773 - 48146) |
| Number of female participants or respondents |  |
| BMI measurement | 22717 (10551 - 51824) |
| Systolic BP measurement | 22728 (10537 - 51806) |
| Diastolic BP measurement | 22707 (10550 - 51822) |
| Smoking status | 22749 (10583 - 51828) |
| Alcohol drinking status | 20479 (9689 - 49216) |
| Amount of alcohol consumption per drinking day | 12312 (5736 - 31628) |
| BMI, body mass index; BP, blood pressure |  |

Supplementary table 2. Number of participants for each type of measurement by deprivation level.

| Outcomes | Quintile 1 (Least deprived areas) | Quintile 2 | Quintile 3 | Quintile 4 | Quintile 5 (Most deprived areas) | | |
| --- | --- | --- | --- | --- | --- | --- | --- |
| Men |  |  |  |  |  | | |
| Systolic BP | 4,109,783 | 4,201,093 | 3,779,479 | 2,342,390 | 1,109,731 | | |
| Diastolic BP | 4,109,488 | 4,200,620 | 3,779,299 | 2,342,146 | 1,109,495 | | |
| BMI | 4,108,952 | 4,198,695 | 3,778,777 | 2,341,536 | 1,108,832 | | |
| Smoking | 4,109,446 | 4,200,164 | 3,779,381 | 2,342,931 | 1,111,470 | | |
| Alcohol drinking frequency | 3,820,241 | 3,854,001 | 3,490,401 | 2,107,814 | 994,427 | | |
| Alcohol drinking amount | 3,203,902 | 3,331,770 | 2,896,082 | 1,739,069 | 823,432 | | |
| Women |  |  |  |  |  | | |
| Systolic BP | 3,337,218 | 3,525,024 | 3,239,244 | 2,037,527 | 1,010,005 | | |
| Diastolic BP | 3,336,977 | 3,524,580 | 3,238,821 | 2,037,253 | 1,009,531 | | |
| BMI | 3,336,554 | 3,524,586 | 3,238,833 | 2,037,104 | 1,009,661 | | |
| Smoking | 3,338,109 | 3,526,529 | 3,239,705 | 2,038,848 | 1,012,089 | | |
| Alcohol drinking frequency | 3,121,553 | 3,255,137 | 3,031,389 | 1,851,822 | 919,926 | | |
| Alcohol drinking amount | 2,149,779 | 2,423,413 | 2,016,952 | 1,214,906 | 603,389 | | |
| BMI, body mass index; BP, blood pressure | | | | | |  |  |

Supplementary table 3. Sex and age group–specific proportion of each of the outcomes in all of Japan.

|  | Age group | | | | | | |
| --- | --- | --- | --- | --- | --- | --- | --- |
| Variable | 40–44 | 45–49 | 50–54 | 55–59 | 60–64 | 65–69 | 70–74 |
| Men |  |  |  |  |  |  |  |
| Proportion of persons whose systolic BP ≥ 140 (mmHg) | 10.2 | 13.6 | 17.2 | 20.9 | 25.2 | 28.3 | 29.6 |
| Proportion of persons whose diastolic BP ≥ 90 (mmHg) | 12.0 | 16.8 | 20.1 | 21.0 | 19.1 | 14.8 | 10.9 |
| Proportion of persons whose BMI ≥ 25 (kg/m^2^) | 34.9 | 37.5 | 37.9 | 36.2 | 34.3 | 31.8 | 28.6 |
| Proportion of persons whose BMI ≥ 30 (kg/m^2^) | 7.6 | 8.0 | 7.2 | 5.7 | 4.3 | 3.1 | 2.3 |
| Proportion of smokers | 40.3 | 39.0 | 37.1 | 34.6 | 31.3 | 24.6 | 18.0 |
| Proportion of drinkers | 67.1 | 68.5 | 70.7 | 72.5 | 72.7 | 70.8 | 67.8 |
| Proportion of heavy alcohol drinkers | 24.1 | 25.4 | 27.2 | 27.8 | 25.7 | 20.5 | 15.1 |
| Women |  |  |  |  |  |  |  |
| Proportion of persons whose systolic BP ≥ 140 (mmHg) | 4.3 | 7.3 | 10.9 | 13.5 | 18.0 | 23.4 | 27.3 |
| Proportion of persons whose diastolic BP ≥ 90 (mmHg) | 4.4 | 6.6 | 8.6 | 8.9 | 8.9 | 8.1 | 7.1 |
| Proportion of persons whose BMI ≥ 25 (kg/m^2^) | 17.5 | 19.8 | 20.7 | 20.5 | 21.3 | 21.8 | 22.1 |
| Proportion of persons whose BMI ≥ 30 (kg/m^2^) | 4.7 | 5.0 | 4.8 | 4.2 | 3.8 | 3.3 | 3.0 |
| Proportion of smokers | 13.2 | 13.7 | 12.1 | 11.1 | 8.6 | 5.4 | 3.6 |
| Proportion of drinkers | 48.8 | 49.3 | 47.4 | 44.9 | 39.5 | 32.7 | 27.8 |
| Proportion of heavy alcohol drinkers | 31.7 | 32.2 | 30.2 | 27.6 | 22.0 | 15.0 | 10.7 |
| BMI, body mass index; BP, blood pressure | | | | | | | |
